# Differential Diagnosis of Regular Narrow Complex Rhythm During Catheter Ablation for Atrioventricular (AV) Nodal Reentrant Tachycardia

**DOI:** 10.7759/cureus.97080

**Published:** 2025-11-17

**Authors:** Moustafa Haroun, Peter Kabunga

**Affiliations:** 1 Cardiology, Darent Valley Hospital, Dartford, GBR; 2 General Medicine, Good Hope Hospital, Birmingham, GBR; 3 Cardiology, King’s College Hospital, London, GBR

**Keywords:** ep, junctional tachycardia, narrow complex tachycardia, premature atrial complex, slow-fast avnrt

## Abstract

Differentiating atrioventricular nodal reentrant tachycardia (AVNRT) from non-reentrant junctional tachycardia during ablation is essential to avoid unnecessary catheter ablation and to minimize the risk of complete atrioventricular (AV) block. We report the case of a 51-year-old woman with symptomatic narrow-complex tachycardia who was referred for electrophysiological study and ablation. Typical AVNRT was induced and ablated successfully at the slow pathway region. Shortly afterward, a spontaneous regular narrow-complex rhythm (NCR) emerged, mimicking recurrent AVNRT. Careful analysis showed that premature atrial complexes (PACs) advanced the His potential without interrupting the NCR, suggesting a junctional rhythm rather than a recurrence of AVNRT. Overdrive pacing from the proximal coronary sinus produced an A-H-H-A response, confirming the junctional origin. This case highlights the diagnostic challenge of distinguishing recurrent AVNRT from junctional rhythm after ablation, as junctional rhythms may masquerade as recurrent AVNRT during postablation monitoring; therefore, careful interpretation of key maneuvers, including the response to PACs and overdrive pacing, is crucial for accurate diagnosis, prevention of unnecessary repeat ablation, and reduction of AV block risk.

## Introduction

Atrioventricular nodal reentrant tachycardia (AVNRT) is the most common type of paroxysmal supraventricular tachycardia (SVT) and is frequently managed with slow pathway catheter ablation [1]. While this approach is highly effective, it may occasionally give rise to regular narrow-complex tachycardias, creating a diagnostic dilemma in distinguishing recurrent AVNRT from junctional tachycardia (JT). Making this distinction is critical: recurrent AVNRT usually indicates incomplete ablation, whereas JT is typically a benign consequence of the procedure. Misinterpreting JT as recurrent AVNRT can result in unnecessary additional ablation, thereby increasing the risk of atrioventricular (AV) block [2]. Electrophysiological (EP) maneuvers aid in this distinction. Padanilam et al. demonstrated that atrial extrastimulus pacing can differentiate JT from AVNRT by showing His advancement without tachycardia termination [3]. Fan et al. described atrial overdrive pacing, where an A-H-H-A response favors JT, whereas an A-H-A response supports AVNRT [4]. More recent literature continues to highlight the importance of structured pacing maneuvers [5]. We report a case of a postablation narrow-complex rhythm (NCR) mimicking recurrent AVNRT, which was ultimately confirmed as JT through pacing maneuvers, thereby avoiding unnecessary further ablation.

## Case presentation

**Figure 1:**
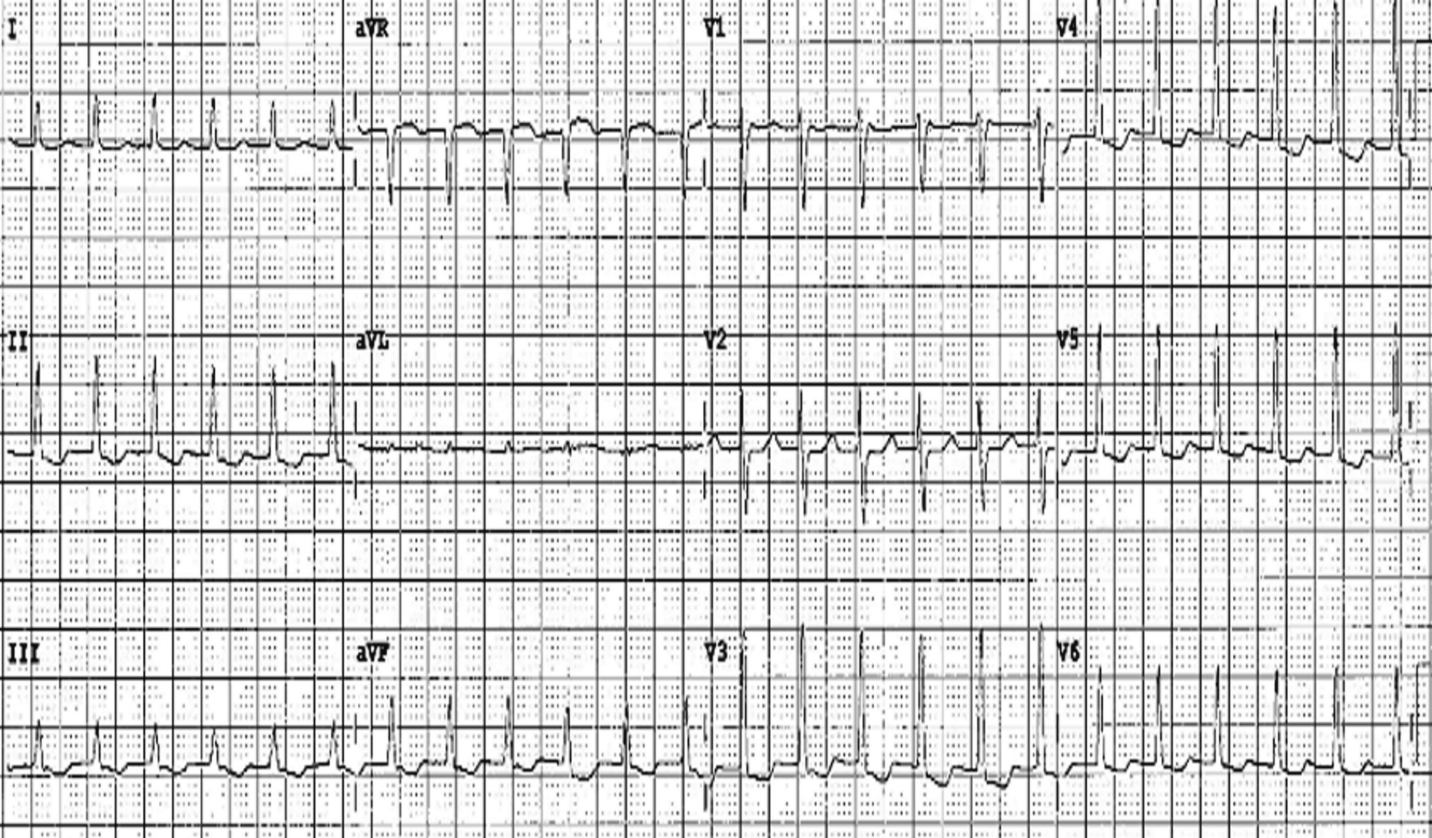
Twelve-lead ECG during the tachycardia aVF: augmented vector foot; aVL: augmented vector left; aVR: augmented vector right; V1-V6: ventricular leads 1-6

A 51-year-old woman with no significant structural heart disease presented with a 10-year history of recurrent, sudden-onset palpitations associated with light-headedness and occasional presyncope, but no chest pain or dyspnea. Episodes were paroxysmal, lasting from a few minutes to over an hour, and were abruptly terminated by vagal maneuvers on several occasions. There was no history of stimulant use, thyroid disease, or prior cardiac surgery. Her physical examination was unremarkable between episodes, and her ECG during the episode is consistent with SVT (Figure 1). Baseline transthoracic echocardiography revealed normal left ventricular function and no valvular abnormalities. Owing to symptomatic recurrences despite beta-blocker therapy, the patient was electively admitted for an EP study and catheter ablation.

**Figure 2:**
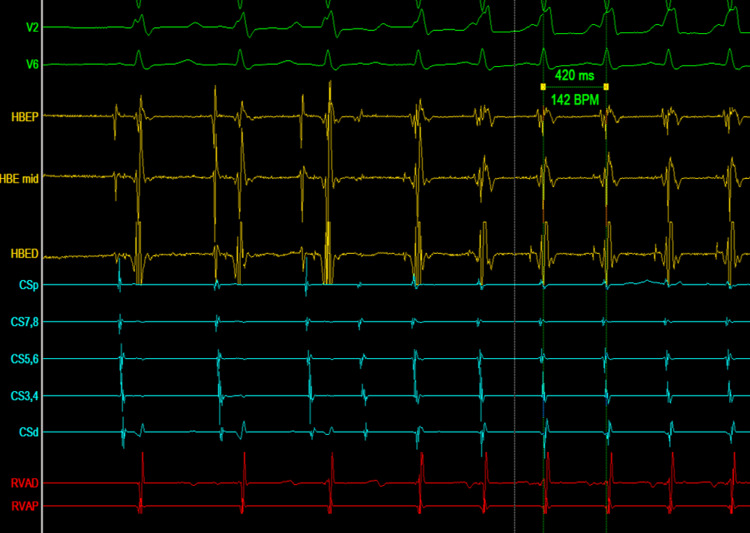
Tachycardia initiated by a PAC and A-H prolongation is seen with a TCL of 420 ms, and rate-related bundle branch block is observed BPS: beats per minute; CS: coronary sinus; CSd: coronary sinus distal; CSp: coronary sinus proximal; HBE: His bundle electrogram; HBED: His bundle electrogram distal; HBEP: His bundle electrogram proximal; RVAD: right ventricular apex distal; RVAP: right ventricular apex proximal; V2, V6: surface ECG leads; PAC: premature atrial complex; TCL: tachycardia cycle length

During the EP study, spontaneous tachycardia was initiated by a premature atrial complex (PAC) accompanied by A-H interval prolongation. The tachycardia cycle length was 420 ms, and a rate-related bundle branch block was observed. The findings were consistent with typical slow-fast AVNRT (Figure 2). Consequently, radiofrequency ablation was performed in the standard slow-pathway region, resulting in apparent acute procedural success.

**Figure 3:**
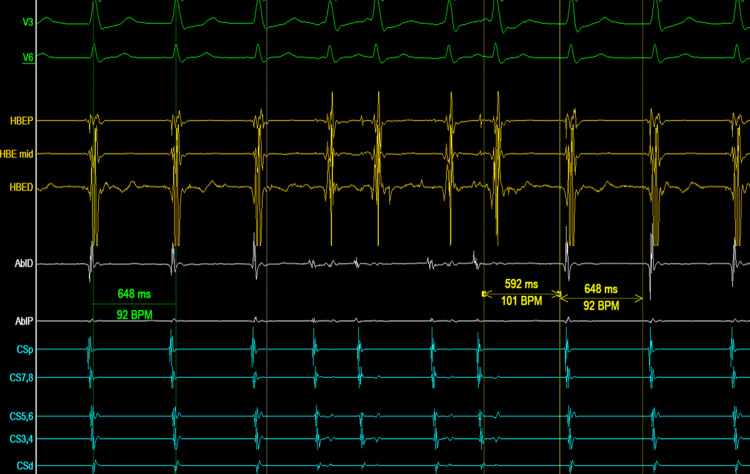
Spontaneous PACs are seen during a regular NCR. The His potential following the PACs is advanced without terminating the NCR AblD: ablation electrode distal; AblP: ablation electrode proximal; BPM: beats per minute; CSd: coronary sinus distal; CSp: coronary sinus proximal; HBE: His bundle electrogram; HBED: His bundle electrogram distal; HBEP: His bundle electrogram proximal; V3, V6: surface ECG leads; PACs: premature atrial complexes; NCR: narrow-complex rhythm

Ten minutes into the waiting period, a spontaneous NCR suggestive of a junctional origin was recorded. Spontaneous PACs were recorded during this rhythm, and notably, the His potential following the PACs was advanced without terminating the narrow-complex tachycardia (Figure 3). This observation suggests that the retrograde fast pathway is not essential for the circuit and that this was a junctional rhythm and not a recurrence of AVNRT, as no dual response of the AV node was seen during the EP study [3].

**Figure 4:**
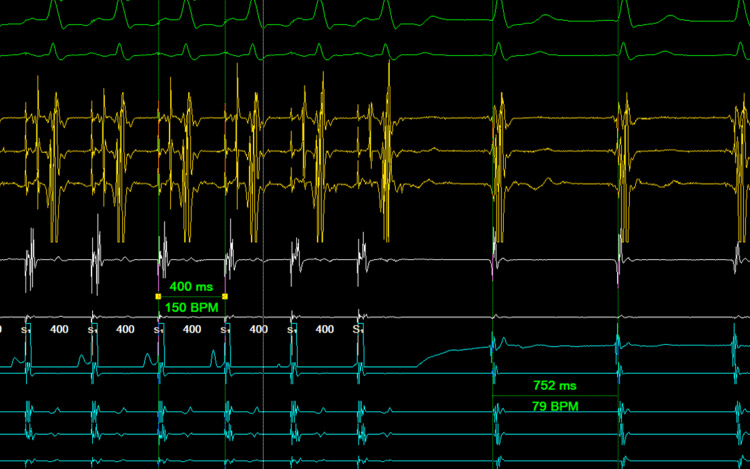
Entrainment from the proximal coronary sinus at 400 ms reveals an A-H-H-A return response BPM: beats per minute

Following atrial overdrive pacing from the proximal coronary sinus, an A-H-H-A response was seen, confirming that the NCR was junctional in origin (Figure 4).

An A-H-A response would be expected if this were a recurrence of AVNRT [4]. At the nine-month follow-up, the patient continues to be symptom-free without the need for medical therapy. A repeat EP study will be pursued if there is any recurrence of tachycardia.

## Discussion

This case illustrates the diagnostic challenges of distinguishing recurrent AVNRT from JT following slow pathway ablation. Both present as regular NCRs, but their implications differ significantly. In our patient, PACs advanced the His potential without terminating the tachycardia, excluding AVNRT and suggesting a junctional origin, consistent with the observations of Padanilam et al. [3]. Overdrive pacing yielded an A-H-H-A response, a hallmark of JT as reported by Fan et al. [4]. The concordance of these findings provided robust evidence against recurrent AVNRT.

Misinterpreting JT as a recurrence of AVNRT can result in unnecessary ablation, increasing the risk of complete AV block. As noted in prior JACC reports, such iatrogenic complications can be avoided by careful pacing analysis [3,5]. Our patient remained asymptomatic and arrhythmia-free at nine months, underscoring the importance of accurate diagnosis and conservative management when appropriate. Previous series have highlighted the diagnostic utility of PAC response and pacing maneuvers [3,4], and more recent studies reaffirm their role in the postablation setting [5].

## Conclusions

Postablation JT can resemble recurrent AVNRT. Therefore, careful assessment of the PAC response and overdrive pacing is essential to establish an accurate diagnosis, prevent unnecessary ablation, and minimize the risk of AV block.
